# Suicidal Ideation and Skill Use During In-patient Dialectical Behavior Therapy for Borderline Personality Disorder. A Diary Card Study

**DOI:** 10.3389/fpsyt.2018.00152

**Published:** 2018-04-20

**Authors:** Thomas Probst, Verena Decker, Eva Kießling, Sascha Meyer, Christine Bofinger, Günter Niklewski, Andreas Mühlberger, Christoph Pieh

**Affiliations:** ^1^Department for Psychotherapy and Biopsychosocial Health, Danube University Krems, Krems an der Donau, Austria; ^2^Institute for Psychology, Regensburg University, Regensburg, Germany; ^3^Department of Psychiatry and Psychotherapy, Paracelsus Medical University, Nuremberg, Germany

**Keywords:** dialectical behavior therapy, borderline personality disorder, in-patient treatment, skill use, suicidal ideation

## Abstract

Associations between suicidal ideation and skill use were investigated during in-patient dialectical behavior therapy (DBT) for borderline personality disorder (BPD). Participants were *N* = 44 patients with BPD undergoing a 5-week in-patient DBT program in a psychiatric clinic. They filled in a diary card each treatment day resulting in 1,334 skill use ratings and 1,364 suicidal ideation ratings. Treatment days were categorized as days with successful skill use (using skills and perceiving them as effective), days with no skill use, days with unsuccessful skill use (using skills but perceiving them as ineffective). Multilevel models were performed to account for the nested data structure. The results showed that suicidal ideation improved more for patients who applied skills successfully more often during treatment (*p* < 0.05). Moreover, suicidal ideation was lower on treatment days with successful skill use compared to treatment days with no skill use and compared to treatment days with unsuccessful skill use (*p* < 0.05). When treatment days with no skill use were compared to treatment days with unsuccessful skill use, suicidal ideation was higher on treatment days with unsuccessful skill use (*p* < 0.05). To conclude, using skills successfully on as many treatment days as possible is associated with lower suicidal ideation.

## Introduction

Borderline personality disorder (BPD) has prevalence rates ranging between 0.5 and 5.9% in the general population [1] and is associated with high health care costs (e.g., [2]). Meta-analyses and reviews reported that patients with BPD benefit from psychotherapies (e.g., [3, 4]). Psychotherapies are also cost-effective treatments of BPD (e.g., [5, 6]). One psychotherapy for patients with BPD is “dialectical behavior therapy” (DBT; [7]). Several interventions are applied in DBT such as validation, telephone coaching, and skills training. The basic skills training comprises mindfulness, interpersonal effectiveness, emotion regulation, and distress tolerance skills [7]. Self-esteem skills or skills to manage addiction can be added to this basic set [8].

Previous studies on outpatient DBT explored whether skill use is associated with an improved treatment outcome. Stepp et al. [9] investigated 27 participants (17 of them with a BPD diagnosis) receiving on average 49 weeks of outpatient DBT and found that overall skill use was significantly correlated with a reduction of borderline features (measured with the “Personality Assessment Inventory-Borderline Features Scale,” PAI-BOR; [10]). In more detail, patients with more overall skill use showed more benefits on the PAI-BOR total score, and the PAI-BOR subscales affective instability, identity disturbance, as well as negative relationship. Neacsiu et al. [11] re-analyzed data of 108 women with BPD participating in previous randomized controlled trials on outpatient DBT (treatment duration: 1 year). They reported that skill use mediated the decrease of several symptoms such as suicide attempts and depression when DBT and the control treatment were analyzed together. In another study, Barnicot et al. [12] explored how skill use, the therapeutic alliance, treatment credibility, and self-efficacy are related to treatment dropout and self-harm in 70 patients with BPD during 1-year outpatient DBT. The results of their study showed that skill use is the most important predictor (compared to the other investigated variables) of dropout and that more skill use as well as higher self-efficacy were independently associated with less self-harm. Linehan et al. [13] performed a randomized clinical trial to isolate the effect skills training exerts on outcomes of DBT. Three DBT conditions were compared against each other. Two conditions included skills training (skills training and case management; skills training and individual therapy) and one condition did not include skills training (individual therapy and activities group). The patients receiving the DBT condition without skills training showed several less favorable outcomes (non-suicidal self-injury, depression, anxiety, dropout rates, use of crisis services, psychiatric hospitalizations) compared to the patients receiving a DBT condition including skills training. In another trial on skills training, DBT skills training was compared with standard group therapy in patients with BPD [14]. The standard group therapy had a less favorable outcome than DBT skills training. All the studies cited in the passage above rely on outpatient DBT. However, patients with BPD are frequently admitted to in-patient treatments [15]. Swenson et al. [16] described how DBT can be applied in in-patient settings. In a review, Bloom et al. [15] found 11 studies on in-patient DBT for BPD. Most of these studies evaluated the treatment outcome so that “we cannot draw conclusions about which components of inpatient DBT contribute to clinical improvement” ([15], p. 886). The present study investigated how skill use is associated with suicidal ideation during in-patient DBT for BPD. Suicidal ideation is an important treatment target in DBT and suicidal ideation can make admissions to in-patient units necessary [17]. Therefore, Bloom et al. [15] recommended investigating suicidal ideation in studies on in-patient DBT for BPD. Suicidal ideation is a common symptom of patients with BPD and can result in suicidal behavior: 60–70% of the patients with BPD make a suicide attempt and death/mortality by suicide ranges between 8 and 10% [17]. The ideation-to-action framework with its conceptual separation between suicidal ideation and suicidal behavior is important in this context [18, 19]. Linehan [20] proposed an emotion dysregulation theory of suicide. Genetic vulnerabilities and environmental factors contribute to emotion dysregulation and the development of BPD in general according to the biosocial developmental model [20, 21]. Emotion dysregulation as well as suicidal ideation, and suicidal behavior can be effectively treated with DBT (e.g., [7, 22, 23]).

According to previous studies showing positive associations between skill use and outcomes in outpatient DBT [9, 11–14], the hypotheses of the study at hand were as follows:

1) Suicidal ideation improves more for patients with more treatment days with successful skill use.

2) Suicidal ideation is lower on treatment days with successful skill use compared to treatment days with no skill use and compared to treatment days with unsuccessful skill use.

We also compared suicidal ideation between treatment days with unsuccessful skill use and treatment days with no skill use, but we had no specific hypothesis here. *Successful* skill use means using skills and experiencing them as helpful, *unsuccessful* skill use means using skills and experiencing them as ineffective, and *no* skill use means not using skills.

## Methods

The present study retrospectively analyzed diary cards. The diary cards are part of the clinical routine and the diary cards were not administered for study purposes. No written informed consent to take part in a study administering and analyzing the diary cards was, therefore, obtained. The patients gave, however, informed consent to take part in a study evaluating the in-patient DBT with questionnaires. The study was approved by the study center of the Clinic for Psychiatry and Psychotherapy, Nuremberg Hospital North, Germany. All the data were saved anonymously.

### Participants

Patients were treated between September 2015 and August 2016 at the unit for psychiatric crisis intervention (20 IV links) of the Clinic for Psychiatry and Psychotherapy at the Nuremberg Hospital North (Germany). Only patients with a BPD diagnoses entered the 5-week in-patient DBT program. The diagnostic procedure to make the BPD diagnoses was a combination of the diagnostics of the referring practitioners, the diagnostics of the therapeutic staff at the unit for psychiatric crisis intervention (20 IV links) of the Clinic for Psychiatry and Psychotherapy at the Nuremberg Hospital North (Germany), and the application of the “International Personality Disorder Examination” (IPDE; [24]). The *N* = 44 participating patients were on average *M* = 30.16 (*SD* = 9.39) years old. Thirty-three patients were female (75%) and 11 patients were male (25%). The symptom severity at intake—measured with the short version of the “Borderline Symptom List” (BSL-23; [25])—amounted to *M* = 1.90 (*SD* = 0.89) and this average value is similar to the BSL-23 mean of *M* = 2.05 found by Bohus et al. [25] in a different sample of patients with BPD. The therapeutic staff assessed psychiatric comorbidity and six patients had one additional psychiatric disorder, 9 patients had two additional psychiatric diagnoses, and two patients had three additional psychiatric diagnoses. The two most frequent comorbidities were diagnoses according to ICD-10 “F4: Neurotic, stress-related, and somatoform disorders” (*n* = 8) and “F1: Mental and behavioral disorders due to psychoactive substance use” (*n* = 7).

### Treatment

The 5-week in-patient DBT program at the unit for psychiatric crisis intervention (20 IV links) of the Clinic for Psychiatry and Psychotherapy at the Nuremberg Hospital North (Germany) is certified by the “Dachverband Dialektisch Behaviorale Therapie (DDBT).” The weekly treatment plan is illustrated in Table 1. In addition, the patients received individual psychotherapy twice a week, individual contacts with the responsible nurse once or twice a week, and individual body psychotherapy once a week. In each 5-week in-patient DBT program, a closed group up to six patients participates. During the time period of the current study, nine groups took part in the 5-week in-patient DBT program: One group consisted of three patients, two groups comprised four patients, two groups included five patients, and four groups had six patients. The group sizes differed from each other because the psychiatric clinic started the 5-week in-patient DBT program at specific months (during the period of this study: September, October, November, January, February, April, May, June, July) and the group size depended on the amount of registrations for each month.

**Table 1:** Weekly treatment plan of the 5-week in-patient DBT program.

**Monday**	**Tuesday**	**Wednesday**	**Thursday**	**Friday**
9.10–max. 9.30: Morning round	9.10–max. 9.30: Morning round	9.10–max. 9.30: Morning round	9.10–max. 9.30: Morning round	9.10–max. 9.30: Morning round
9.45–10.30: Patient group without therapists	9.45–11.15: Interpersonal effectiveness skills	9.30–11: Skills training in general		9.45–10.30: Patient group without therapists
11.30–13: Skills training in general	11.30–13: Consultation team (therapists only)			11–11.30: Mindfulness skills
Lunch	Lunch	Lunch	Lunch	Lunch
	12.45–13.30: Physical exercise (v)	12.45–13.30: Physical exercise (v)	12.45–13.30: Psychoeducation	12.30–13.15: Imagination (v)
	13.00–14.30: Art therapy ^*^			
15–16: Anxiety management (v)	14.30–16: Creative work^*^	14.30–16: Handicrafts ^*^	14.45–16.15: Ergotherapy (v)	
16.30–17.15: Acupuncture (v)		16.30–17: Acupuncture (v)	15.30–16.15: Sleep hygiene (v)	16.30–17.15: Acupuncture (v)
Approximately 18: Individual contact to analyze the diary card (5–10 min)	Approximately 18: Individual contact to analyze the diary card (5–10 min)	Approximately 18: Individual contact to analyze the diary card (5–10 min)	Approximately 18: Individual contact to analyze the diary card (5–10 min)	Approximately 18: Individual contact to analyze the diary card (5–10 min)
		18–19.30: Ergotherapy (v)		

V, voluntary;

* *Mandatory participation in at least one of these treatments; not shown in the table are the individual appointments for each patient including 2 individual psychotherapy sessions (30 min each) per week, 1–2 individual sessions with the responsible nurse (~30 min each) per week, and 1 individual body psychotherapy session (30 min) per week; while there are specific groups for the interpersonal effectiveness skill module and the mindfulness skill module, the other skill modules (emotion regulation, distress tolerance) are covered by other group therapies (e.g., skills training in general) and individual therapy*.

### Measures

The patients filled in a diary card at each treatment day during the 5-week in-patient DBT program. The following two variables of the diary card were analyzed in the present study:

- Suicidal ideation: The patients rated their suicidal ideation on the diary card for each treatment day on a 6-point scale from 0 (min) to 5 (max) with the options “no” (0), “slightly” (1), “moderate” (2), “urgent” (3), “very urgent” (4), and “thinking is completely narrowed to suicidal ideation” (5).
- Skill use: The patients rated their skill use for each treatment day. There were eight options on the diary card: “did not think about using skills and did not use them” (0), “thought about it, but did not use, did not want to” (1), “thought about it, did not use, but wanted to use” (2), “tried to use, but could not use skills” (3), “tried to use, could use skills, but they did not help” (4), “used skills automatically, but they did not help” (5), “tried to use, could use skills, and they helped” (6), “used skills automatically and they helped” (7). In case a patient gave a rating between these skill use categories of the diary card, the rating was assigned to the more conservative category (e.g., a rating between the categories 6 and 7 was assigned to category 6).

### Statistics

All statistical analyses were performed with SPSS 24. Frequencies (*n, %*), means (*M*), and standard deviations (*SD*) were calculated for the sample description.

To evaluate the first hypothesis, we analyzed whether suicidal ideation improves stronger for patients with more treatment days with successful skill use. Skill use was dichotomized for these analyses. The diary card ratings 0–5 were defined as no-successful skill use and the ratings 6–7 were defined as successful skill use.

Two linear multilevel models with two levels (level 1: days; level 2: patients) were performed. Suicidal ideation was the dependent variable in both models and the time variable (day; first day set to 0) was the predictor. To handle missing data of the outcome variable, the full maximum likelihood estimation was applied in both models. Moreover, an unstructured variance-covariance matrix (allowing the intercept and the slope to vary randomly) was selected in both models.

The first multilevel model investigated whether the *frequency* of treatment days with successful skill use is associated with an improved course of suicidal ideation during in-patient DBT. The sum of days with successful skill use was calculated for each patient to obtain his/her *frequency* of treatment days with successful skill use. This model investigated the following main and interaction effects: intercept, intercept ^*^ frequency of days with successful skill use, slope, slope ^*^ frequency of days with successful skill use. “*Frequency* of days with successful skill use” was added as z-standardized time-invariant covariate.

The second multilevel model evaluated whether the *percentage* of treatment days with successful skill use correlates with improvements in suicidal ideation during in-patient DBT. This second model was performed to validate the results of the first multilevel model, since patients filling in more diary cards can *per se* have a higher frequency (but not percentage) of days with successful skill use than patients with more missing diary cards. To obtain a patient's *percentage* of treatment days with successful skill use, his/her sum of days with successful skill use (frequency of days with successful skill use) was divided through his/her sum of days with a skill use diary card entry. This model evaluated the following main and interaction effects: intercept, intercept ^*^ percentage of days with successful skill use, slope, slope ^*^ percentage of days with successful skill use. “*Percentage* of days with successful skill use” was added as z-standardized time-invariant covariate to the model.

To investigate the second hypothesis, we analyzed whether suicidal ideation was lower on treatment days with successful skill use compared to treatment days with no skill use and compared to treatment days with unsuccessful skill use. Another linear multilevel model with two levels (level 1: days; level 2: patients) was performed. The diary card entries for skill use were recoded as follows to define successful, unsuccessful, and no skill use: ratings 6–7 (used skills and they helped) = successful skill use; ratings 4–5 (used skills, but they did not help) = unsuccessful skill use; ratings 0–3 (no use of skills) = no skill use. In this multilevel model, this recoded skill use variable was added as factor and suicidal ideation was the dependent variable. *Post-hoc* tests with Bonferroni correction were applied to contrast the three categories of skill use against each other. Again, the multilevel model was performed with the full maximum likelihood estimation to manage missing data of the outcome variable.

All statistical tests were performed two-tailed with a significance value of *p* < 0.05.

## Results

A maximum of 1,540 (44 patients ^*^ 35 treatment days) diary cards would have been possible. For the variable skill use, 1,334 diary card entries were available (86.6%) and 1,364 diary card entries were available for the variable suicidal ideation (88.6%). Patients used skills and experienced them as helpful on most of the days during in-patient DBT, since 1,064 diary card entries (79.8% of 1,334) indicated successful skill use. On average, the *N* = 44 patients performed skills successfully on *M* = 24.18 (*SD* = 8.97) days during the 35 in-patient DBT days. Moreover, the *N* = 44 patients provided diary card entries on skill use on average for *M* = 30.32 days (*SD* = 5.97) during the 35 in-patient DBT days. The average ratio of “successful skill use/diary card entries on skill use” was *M* = 79.6% (*SD* = 23.1%).

Table 2 presents the results of the multilevel model addressing the question whether a higher *frequency* of treatment days with successful skill use is associated with an improved course of suicidal ideation during in-patient DBT. Table 3 summarizes the results of the multilevel model evaluating whether a higher *percentage* of treatment days with successful skill use correlates with improvements in suicidal ideation during in-patient DBT. Results were comparable in both models. Suicidal ideation improved more for patients with a higher frequency/percentage of treatment days with successful skill use (negative “slope ^*^ frequency/percentage of days with successful skill use” term; *p* < 0.05). Moreover, suicidal ideation at the first treatment day was not associated with the frequency/percentage of skill use (“Intercept ^*^ frequency/percentage of days with successful skill use” term; *p* > 0.05).

**Table 2:** Results of the multilevel model on associations between *frequency* of days with successful skill use and course of suicidal ideation during in-patient DBT (“frequency of days with successful skill use” z-standardized).

**Suicidal Ideation**	**Parameter**	**Estimate (SE)**	**Statistics**
Fixed effects	Intercept	0.723 (0.137)	*t*_(44.00)_ = 5.28; *p* < 0.001
	Intercept ^*^ frequency of days with successful skill use	−0.095 (0.139)	*t*_(44.10)_ = −0.68; *p* = 0.498
	Slope	0.007 (0.004)	*t*_(39.94)_ = 1.77; *p* = 0.085
	Slope ^*^ frequency of days with successful skill use	−0.015 (0.004)	*t*_(40.23)_ = −3.49; *p* = 0.001
Random effects	Residual	0.468 (0.019)	Wald *Z* = 25.25; *p* < 0.001
	Intercept	0.765 (0.176)	Wald *Z* = 4.36; *p* < 0.001
	Intercept ^*^ slope	−0.009 (0.004)	Wald *Z* = −2.26; *p* = 0.024
	Slope	0.0005 (0.0002)	Wald *Z* = 3.39; *p* = 0.001

**Table 3:** Results of the multilevel model on associations between *percentage* of days with successful skill use and course of suicidal ideation during in-patient DBT (“percentage of days with successful skill use” z-standardized).

**Suicidal Ideation**	**Parameter**	**Estimate (SE)**	**Statistics**
Fixed effects	Intercept	0.731 (0.133)	*t*_(44.07)_ = 5.51; *p* < 0.001
	Intercept ^*^ percentage of days with successful skill use	−0.227 (0.134)	*t*_(43.73)_ = −1.70; *p* = 0.096
	Slope	0.006 (0.004)	*t*_(38.72)_ = 1.33; *p* = 0.193
	Slope ^*^ percentage of days with successful skill use	−0.010 (0.004)	*t*_(36.99)_ = −2.41; *p* = 0.021
Random effects	Residual	0.469 (0.019)	Wald *Z* = 25.23; *p* < 0.001
	Intercept	0.715 (0.165)	Wald Z = 4.34; *p* < 0.001
	Intercept ^*^ slope	−0.009 (0.004)	Wald *Z* = −2.20; p = 0.028
	Slope	0.0006 (0.0002)	Wald *Z* = 3.41; *p* = 0.001

Next, we explored whether suicidal ideation is lower on treatment days with successful skill use compared to treatment days with no skill use and compared to treatment days with unsuccessful skill use. The multilevel model revealed that suicidal ideation was significantly different between these three categories [*F*_(2;1246.86)_ = 33.54; *p* < 0.05]. Table 4 displays how often the three skill use categories were reported and the estimated marginal means. The results of the Bonferroni-corrected *post-hoc* tests are displayed in Table 5.

**Table 4:** Frequency of days with successful, unsuccessful, and no skill use as well as estimated marginal means of suicidal ideation at days with successful, unsuccessful, and no skill use.

**Skill use category**	**Frequency *n* (%)**	**Suicidal ideation**
		**M (SE)**
No skill use	91 (6.8)	0.953 (0.134)
Unsuccessful skill use	179 (13.4)	1.237 (0.116)
Successful skill use	1,064 (79.8)	0.733 (0.104)

*SE, Standard Error*.

**Table 5:** Results of the *post-hoc* tests comparing suicidal ideation between days with successful, unsuccessful, and no skill use (Bonferroni-corrected).

**(I) Skill use category**	**(J) Skill use category**	**Difference of means (I-J)**	**SE**	***df***	***p***
No skill use	Unsuccessful skill use	−0.284	0.097	1231.06	0.010
	Successful skill use	0.220	0.087	1264.48	0.034
Unsuccessful skill use	No skill use	0.284	0.097	1231.06	0.010
	Successful skill use	0.504	0.062	1234.67	< 0.001
Successful skill use	No skill use	−0.220	0.087	1264.48	0.034
	Unsuccessful skill use	−0.504	0.062	1234.67	< 0.001

*SE, Standard Error*.

As can be seen in Table 5, suicidal ideation was lower on treatment days with successful skill use compared to treatment days with no skill use and compared to treatment days with unsuccessful skill *(p* < 0.05). Moreover, suicidal ideation was higher on treatment days with unsuccessful skill use compared to treatment days no skill use (*p* < 0.05).

## Discussion

This diary card study analyzed associations between suicidal ideation and skill use during in-patient DBT for BPD. As expected in the first hypothesis, a higher frequency/percentage of days with successful skill use (skills are used and experienced as helpful) was associated with an improved course of suicidal ideation during in-patient DBT. This result is in line with previous research on outpatient DBT [9, 11–14]. Regarding in-patient therapy, a study on depression found that the successful application of emotion regulation skills was correlated with the outcome [26, 27]. These results highlight the importance of successful skill use in DBT and other treatments. On average, the patients of the present study used skills successfully on 80% of the in-patient treatment days. Future studies could investigate whether this already relatively high percentage can be further increased when barriers to successful skill use (see [28]) are addressed for example by CD-ROM-based programs [29], mobile applications [30], or virtual reality scenarios [31, 32].

Another result of the study at hand was that suicidal ideation was lower on treatment days with successful skill use compared to treatment days with no skill use and compared to treatment days with unsuccessful skill use. Therefore, the second hypothesis was confirmed as well. We had no specific hypotheses how treatment days with unsuccessful skill use differ from treatment days with no skill use. Suicidal ideation was higher on treatment days with unsuccessful skill use compared to treatment days with no skill use. This cross-sectional analysis does not allow drawing causal inferences, but it could be that skill use without positive consequences increases subsequent suicidal ideation or that high suicidal ideation hinders subsequent successful skill use. To gain a deeper understanding of the temporal relationship between suicidal ideation and skill use, future studies with larger samples are necessary. These studies should also assess skill use and suicidal ideation on several time points during a day to identify potential vicious circles such as “high suicidal ideation at time X→skill use without positive consequences at time X+1→even higher suicidal ideation at time X+2” or potential virtuous circles such as “high suicidal ideation at time X→successful skill use at time X+1→lower suicidal ideation at time X+2.” Another argument for repeated within-day assessments of suicidal ideation might be that suicide underlies circadian rhythms (e.g., [33]). As the between-day assessments of 44 patients in our study already resulted in over 1,000 diary cards, a larger study with repeated within-day assessments should use mobile electronic questionnaires to store and process the data more easily (e.g., [34, 35]).

A major shortcoming of the current study is that no measures of adherence were applied. As a result, it is difficult to conclude to what extent DBT was studied. However, it should also be noted that obtaining adherence ratings is often not feasible in in-patient settings under the conditions of routine care. The certification of the treatment by the “Dachverband Dialektisch Behaviorale Therapie (DDBT)” can be an argument that the treatment was actually DBT. Another limitation is the assessment of suicidal ideation and skills by diary card items. Assessing suicidal ideation and skills by psychometrically sound questionnaires would result in more reliable and more valid results. Moreover, the one item on skill use assessed skills in general and different skills might be more or less important for suicidal ideation. Other studies already reported that some specific skills are more or less beneficial for patients with BPD. For example, Stepp et al. [9] found that mindfulness and emotion regulation skills were associated with decreases of identity disturbance, whereas interpersonal skills and distress tolerance skills did not show these correlations. Regarding mindfulness skills (observing, describing, acting with awareness, accepting without judgment), only increases of accepting without judgment were related to decreases of BPD symptoms in another study [36]. Future studies could use the psychometrically sound “DBT ways of coping checklist” [37, 38]. However, this instrument comprises 59 items and refers to the last 4 weeks, which makes it difficult to apply this instrument to study skills between or even within treatment days. Another possibility might be to add four items, one item for a specific skill (mindfulness, emotion regulation, interpersonal effectiveness, distress tolerance), to the diary card. Besides skill use, other process variables are related to patient progress in DBT for BPD [39, 40] as well as to patient progress during in-patient psychotherapy [41–43]. Therefore, a shortcoming of our study is that we did not evaluative whether skill use or other DBT / in-patient psychotherapy processes are more or less beneficial for suicidal ideation (see for example [12] for a study comparing several variables in outpatient DBT for BPD). The naturalistic design can be seen as a limitation as well because experimental designs comparing a skills condition with a control condition (as done for example by [13, 14]) have a higher internal validity. On the other hand, the naturalistic design is also a strength since the study took place under the conditions of routine practice increasing the external validity. The relatively small sample size, however, limits the generalizability. A strength of the current study is the use of multilevel models, which account for the nested structure of the data. The relatively high completion rate of the diary cards (>85%) can be seen as another positive aspect of this study.

To summarize, the present study investigated for the first time associations between suicidal ideation and skill use during in-patient DBT. Using skills successfully on as many treatment days as possible is associated with lower suicidal ideation.

## Author contributions

TP: Drafted the manuscript and performed the statistical analyses; VD and EK: Revised the manuscript and processed the data; SM, CB, and GN: Revised the manuscript and were involved in treating the patients; AM and CP: Revised the manuscript and contributed to the study design.

### Conflict of interest statement

The authors declare that the research was conducted in the absence of any commercial or financial relationships that could be construed as a potential conflict of interest.
